# *Ballota hirsuta* Benth Arrests the Cell Cycle, Induces Apoptosis and Inhibits the Invasion of MCF-7 and MDA-MB-231 Cell Lines in 2D and 3D Models

**DOI:** 10.3390/ijms26125672

**Published:** 2025-06-13

**Authors:** Diana del Carmen Martínez-Méndez, María de la Luz Sánchez-Mundo, Laura Adriana Ortiz-León, Luis Marat Álvarez-Salas, Víctor Hugo Rosales-García, Jacobo Rodríguez-Campos, María Eugenia Jaramillo-Flores

**Affiliations:** 1 Ingeniería Bioquímica, Escuela Nacional de Ciencias Biológicas (ENCB)-Instituto Politécnico Nacional, Ciudad de Mexico 07738, Mexico; dmartinezm2111@alumno.ipn.mx (D.d.C.M.-M.); adrianaortizleon@gmail.com (L.A.O.-L.); 2 ITS de Las Choapas, Tecnológico Nacional de México, Carretera Las Choapas-Cerro de Nanchital Km 6.0, Col. J. Mario Rosado, Las Choapas 96980, Mexico; l-sanchezm@choapas.tecnm.mx; 3 Centro de Investigación y de Estudios Avanzados del Instituto Politécnico Nacional (CINVESTAV), Ciudad de Mexico 07360, Mexico; lalvarez@cinvestav.mx (L.M.Á.-S.); vrosales@cinvestav.mx (V.H.R.-G.); 4 Unidad de Servicios Analítico y Metodológicos, Av. Normalistas 800, Col. Colinas de la Normal, Guadalajara 44270, Mexico; jarodriguez@ciatej.mx

**Keywords:** cancer, MCF-7, MDA-MB-231, cell cycle, CDK4, caspases, apoptosis, tumor, invasion

## Abstract

Breast cancer is a disease with a high incidence and mortality rate worldwide. There is a growing interest in the search for alternative treatments with a good cytotoxic effect but fewer adverse effects, because paclitaxel and cis-platinum treatments present severe adverse effects. The aim of this study was evaluating the antitumor activity of ethyl acetate extract of *Ballota hirsuta* Benth (EAB) in breast cancer cell lines. The ${IC}_{50}$ of EAB is 49.3 μg/mL and 3.7 μg/mL in 2D and 375 μg/mL and 135 μg/mL in 3D in the MCF-7 and MDA-MB-231 cell lines, respectively. It arrested the cell cycle in the G1 phase and decreased CDK4 activity by 86%, increasing the p53 protein levels. During the in silico analysis, the compounds interacted with the IGF-R1, CDK1, CDK2, TNFR1, MLKL, MMP2, MMP9, E-cadherin and N-cadherin proteins, which are involved in necroptosis, invasion and the cell cycle. It decreased the ATP levels in 3D by 87% at 600 μg/mL in MCF-7 and 99% at 250 μg/mL in MDA-MB-231; induced apoptosis by increasing the activity of caspases-3/7, -8 and -9; inhibited invasion and enhanced the effect of cisplatin and paclitaxel in combination with EAB. The results show the antitumor potential of EAB as a possible adjuvant in breast cancer therapy.

## 1. Introduction

Cancer is one of the leading causes of death worldwide, and among them, breast cancer ranks second in incidence and fourth in mortality in both sexes, although, in women, it ranks first in incidence and mortality [1]. In Mexico, it is the leading cause of cancer death in women, with an incidence of 27.64 per 100,000 inhabitants and a cancer mortality rate of 17.94 in women aged 20 years [2].

This type of cancer originates in breast cells, generating a malignant tumor that initially invades the surrounding tissues, followed by distant organs (metastasis) [3].

The molecular classification of breast cancer divides mammary carcinomas into hormone-dependent tumors (Luminal A, Luminal B and tumors with overexpression of the HER-2 oncogene) and hormone-independent, such as triple-negative tumors like MDA-MB-231, which do not express ER, PR or HER2, leading to a higher risk of recurrence and worse prognosis [4]. The MCF-7 cell line has provided the most data for treating this type of cancer. It has been widely used in estrogen receptor (ER)-positive experiments with a large number of subclones of ER-positive tumors and varying levels of nuclear receptor expression [5]. Each subtype presents unique characteristics of aggressive behavior, high proliferation, invasion, and metastatic potential, which influence treatment and prognosis. For triple-negative subtypes, it is important to investigate aggressive subtypes such as MDA-MB-231 because of their important role in breast cancer morbidity and mortality [6,7,8].

Treatments include surgery, stem cell or bone marrow transplantation, radiotherapy and targeted drug therapy; among which, we find drugs such as Trastuzumab, which is a monoclonal antibody inhibitor of the HER2 (human epidermal growth factor receptor 2) oncogene [9] and Palbociclib, which can inhibit CDK4/6 activity [10]. Immunotherapy with monoclonal antibodies such as Pembrolizumab and Nivolumab inhibit the PD-1 (programmed cell death receptor 1) receptor on tumor cells [11] and chemotherapy [12]. However, all existing treatments cause very serious side effects, in addition to a growing resistance to current anticancer agents, so the search for new compounds with antitumor characteristics is urgent, and therefore, natural products are of great importance due to their cytotoxic activities, such as phenolic compounds that have been shown to have cytotoxic activity against different cancer cell lines [13].

Therefore, the use of extracts or compounds from them represents an excellent option to use the bioactive compounds they contain as adjuvants in the treatment of breast cancer, reducing the doses of drugs that are used today and thereby reducing the side effects. The genus Ballota L. comprises 33–35 species belonging to Lamiaceae, tribe Stachydae, subtribe Ballotae [14]. Phytochemical analyses of *Ballota hirsuta* Benth have identified different compounds like hispanolone, flavonoids (catechin and epicatechin), phenolic acids (gallic acid and 3,4-dihydroxybenzoic acid), polyphenols and tannins responsible for its biological activity with antioxidant and cytotoxic effects, suggesting a role in cancer treatment [15]. Hispanolone derivatives showed that this compound activates caspases-3, -8 and -9 [16]. It has been reported that hispanolone arrests the cell cycle in the G1 phase in glioblastoma cells, thus halting cell division [17].

Most cancer biology research is based on experiments with two-dimensional (2D) in vitro cultures; however, this type of cell culture has limitations, including the inability to replicate interactions between cells and the extracellular environment or maintain cell polarity [18]. These disadvantages are not present in a three-dimensional (3D) culture, which aims to replicate the real tumor environment, resulting in spheroids with genetic and morphological characteristics similar to those of real neoplastic masses. Therefore, this work evaluated the pro-apoptotic activity, invasion inhibition and cell cycle arrest caused by *Ballota hirsuta* Benth extract against the MCF-7 and MDA-MB-231 cell lines in 2D and 3D models.

## 2. Results

### 2.1. Compounds Identification of Ballota hirsuta Benth Extract (EAB) by Ultra-Performance Liquid Chromatography with Time-of-Flight Mass Spectrometry (UPLC-Q-TOF/MS^E^) and the Quantification of Compounds in Crude Extract by HPLC

**Figure 1:**
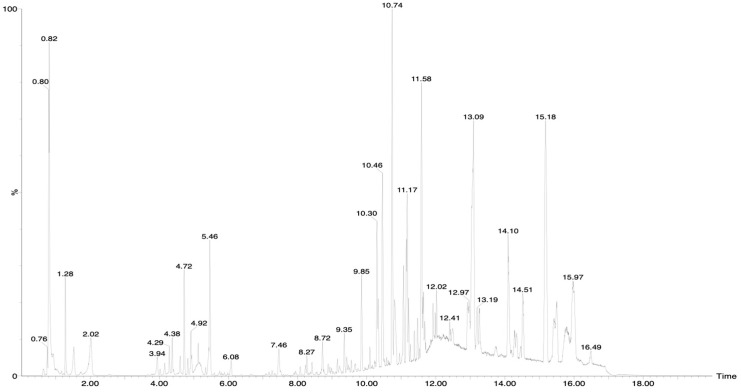
Chemical profile of EAB by Ultra-Performance Liquid Chromatography with Time-of-Flight Mass Spectrometry (UPLC-Q-TOF/MS^E^ Xevo) (G2-XS model, Waters Corporation 34 Maple Street, Milford, MA 01757, USA), Waters ACQUITY UHPLC^®^ HSS T3 (2.1 MM × 100 mm, 1.8 μm particle size). The MS^E^ mode acquisition mode was operated in negative polarity. Experimental conditions: the cone voltage was 15 V, the capillary voltage was 2.5 kV in the negative mode and the desolvation temperature was fixed at 550 °C, while the ion source temperature remained at 12 °C. Desolvation gas (N_2_) flowed at 1000 L/h, while the cone gas (N_2_) flowed at 50 L/h. Data acquisition was on MassLynx software (V4.1. Waters Corporation, Milford, MA, USA).

The UPLC-QTOF-MS^E^ analysis of EAB in negative-ionization mode allowed the visualization of different signals (Figure 1), of which 15 were identified, as shown in Table 1. The compounds observed are phenolic compounds, including tannins that have a polyol nucleus, often glucose esterified with phenolic acids such as gallic acid [19] and flavonoids such as quercetin and epicatechin, which have a 15-carbon flavone skeleton and two benzene rings (A and B) linked by a three-carbon pyran ring [20]. These results show that phenolic compounds are present in EAB.

The amounts of compounds in the ethyl acetate extract determined by HPLC found catechin to be the main metabolite: 4 mg catechin/g dry sample, 1.2 mg epicatechin/g dry sample, 176 µg phloridzin/g dry sample, 82 µg ellagic acid/g dry sample, 23 µg 3,4-dihydroxybenzoic acid/g dry sample and 15 µg gallic acid/g dry sample (Supplementary Table S1).

### 2.2. Two-Dimensional Cell Model

#### 2.2.1. Effect of EAB on the Viability of MCF-7 y MDA-MB-231 Cells by the Sulforhodamine B (SRB) Method

**Figure 2:**
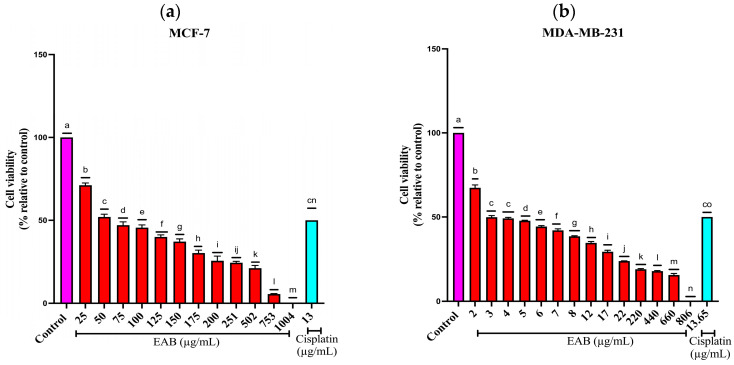
Effect of EAB on the cell viability of breast cancer cells in the 2D model: (**a**) MCF-7 and (**b**) MDA-MB-231. Results are expressed as mean ± SD values. *p* < 0.0001 according to Tukey’s post hoc test, *n* = 12. Different letters represent significant differences between types of treatment.

The EAB extract presented a dose-dependent effect on cell viability in the MCF-7 line, since it decreases as concentrations increase (Figure 2a), with a ${IC}_{50}$ of 49.3 µg/mL, while the cytotoxicity in the MDA-MB-231 cell line was higher, obtaining a ${IC}_{50}$ of 3.7 µg/mL (Figure 2b), higher even than cisplatin. The anticancer activity of the extracts is classified into 4 categories: (A) those extracts with high and selective activity with a ${IC}_{50}$ lower than 10 μg/mL; (B) high and non-selective activity and a ${IC}_{50}$ lower than 10 μg/mL; (C) moderate activity, which has a range of 10–100 μg/mL and (D) no activity [21,22]. According to the above, the effect of EAB on the MCF-7 cell line can be considered moderate activity. In contrast, on the MDA-MB-231 cell line, it exhibits high and selective activity, showing a marked difference between the two cell lines.

#### 2.2.2. Antiproliferative Effect of EAB by Clonogenic Assay in a 2D Model of MCF-7 and MDA-MB-231 Cells

**Figure 3:**
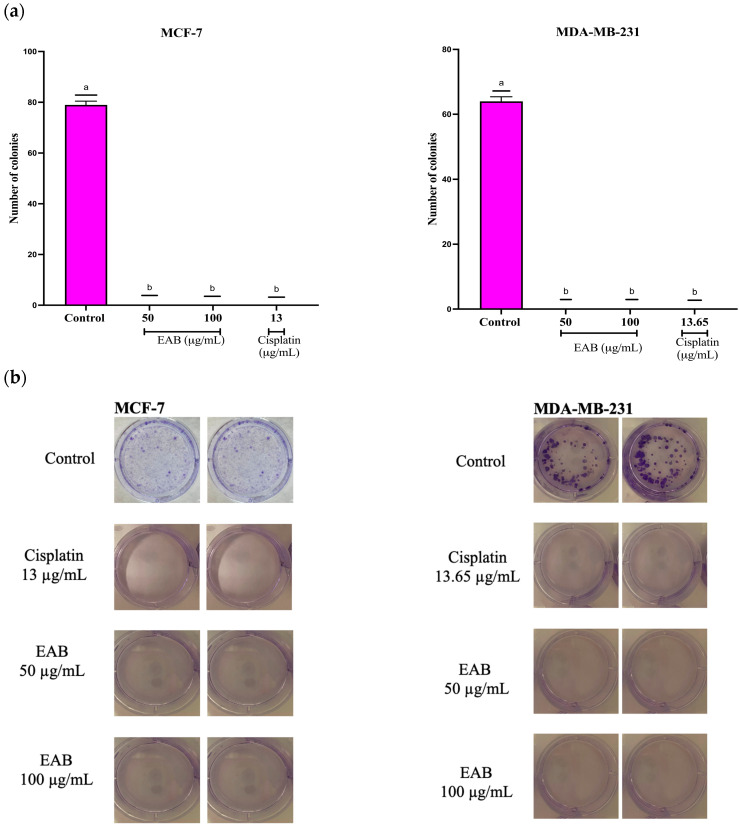
Effect of EAB on colony formation in a 2D cell model in breast cancer cell lines MCF-7 and MDA-MB-231. (**a**) Colony growth analysis after 72 h of exposure to treatment. (**b**) Colony images. Results are expressed as mean ± SD values. *p* < 0.0001 according to Tukey’s post hoc test, *n* = 6. Different letters represent significant differences between types of treatment.

To evaluate the sensitivity of cancer cells to EAB, a clonogenic assay was performed, a method based on the ability of a single cell to divide and form a colony [23], allowing to know the proliferation capacity of the cells after the application of the treatment. As shown in Figure 3, colony formation was not present in both cell lines. This is important, because when a tumor resists treatment and not all neoplastic cells are eliminated, it can promote patient relapse. Drug resistance is a challenge in cancer treatments [24]. Such resistance can be intrinsic, where neoplastic cells show a resistant phenotype or acquired when cancer cells initially respond to treatment and subsequently become insensitive [25,26].

#### 2.2.3. Cell Cycle Arrest in G1 Phase by EAB Treatment in a 2D Model of MCF-7 and MDA-MB-231 Cells

EAB with 500 and 1000 µg/mL arrested most cells in the G1 phase compared to cisplatin, which was arrested in the S phase. This happened in both cell lines; for MCF-7: G1 phase 84%, S 8.3% and G2 7.3% with 500 µg/mL and G1 phase 82%, S 0% and G2 17.8% with 1000 µg/mL; for MDA-MB-231: G1 phase 76.2%, S 8.3% and G2 15.3% with 500 µg/mL and G1 phase 82.85%, S 15.8% and G2 1.3% with 1000 µg/mL (Table 2). These results suggest that the best inhibition occurred in the MDA-MB-231 cell line with 1000 µg/mL, because only 1.3% of the cells were in the G2 phase of the cell cycle.

#### 2.2.4. Inhibition of Cyclin-Dependent Kinase 4 (CDK4) Activity by EAB Treatment

**Figure 4:**
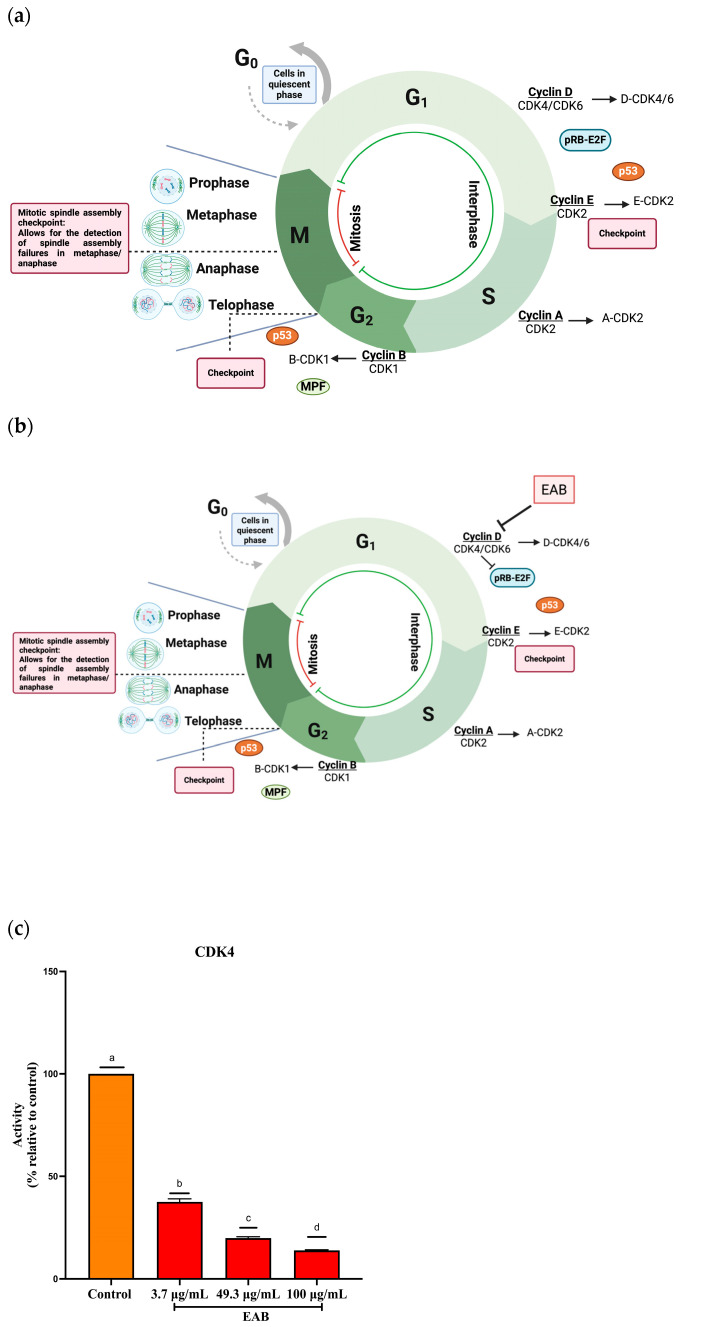
Effect of EAB on cyclin-dependent kinase 4 (CDK4) activity. (**a**) Normal cell cycle. (**b**) Effect of EAB on the cell cycle. (**c**) CDK4 activity at different concentrations of EAB. Results are expressed as mean ± SD values. *p* < 0.0001 according to Tukey’s post hoc test, *n* = 6. Different letters represent significant differences between types of treatment.

As shown in Figure 4c, the activity of this complex decreased when exposed to EAB. This may be due to polyphenols such as catechin and epicatechin, which arrest the cell cycle in the G1 phase by downregulating cyclins D and E [27]. When cyclin is inhibited, the complex is inactivated, and the retinoblastoma tumor suppressor protein (Rb) cannot be phosphorylated; thus, it does not release the transcription factor E2F, and consequently, the transcription of CDK2 and cyclin E necessary for the transition to the S phase of the cell cycle is not induced (Figure 4b) [28].

#### 2.2.5. Increased p53 Protein Activity by EAB Treatment

The normal function of the p53 protein is to control the cell cycle by regulating DNA transcription, which is why it is called the “guardian of the genome”. It is known that two factors can activate the p53 protein (tumor protein p53), the DNA damage and various pro-oncogenic factors [29].

**Figure 5:**
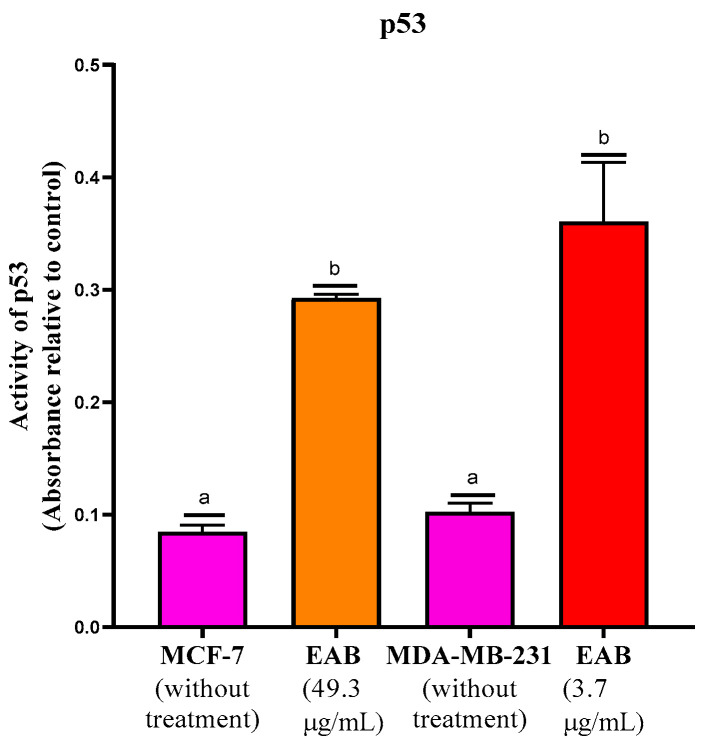
Assay on p53 from EAB-stimulated MCF-7 and MDA-MB-231 cell nuclear extracts. Results are expressed as mean ± SD values. *p* < 0.0001 according to Tukey’s post hoc test, *n* = 6. Different letters represent significant differences between types of treatment.

EAB increased activity in both cell lines compared to the control; however, 3.7 µg/mL was required for the MDA-MB-231 line, whereas 49.3 µg/mL (an extract concentration approximately 16 times higher) of the same extract was required for an activation of equal magnitude in MCF-7 (Figure 5).

### 2.3. Molecular Docking of the Interaction of the Compounds with Proteins Involved in Necroptosis, Cell Invasion and the Cell Cycle

The interaction of proteins involved in necroptosis, cell invasion, the cell cycle and extracellular matrix remodeling was studied. The interaction was performed between ALDH1A3, IGF-R1, CDK1, CDK2, CDK4, p53, TNFR1, MLKL, MMP2, MMP9, E-cadherin and N-cadherin and gallic acid, epigallocatechin, 3,4-dihydroxybenzoic acid, gallic acid 4-O-(6-galloylglucoside), catechin, epicatechin, procyanidin B2, epigallocatechin gallate, gallocatechin 3-gallate, quercetin-3-O-arabinoglucoside, quercetin-3-O-glucoside, ellagic acid, epicatechin-3-O-gallate, quercitrin and phloridzin, of which interactions were found with 8 of the 15 compounds studied, which are shown in Table 3.

The in silico analysis suggests that ALDH1A3 interacts with ellagic acid (free energy −14.04), with 3,4-dihydroxybenzoic acid (free energy −13.74), with catechin (free energy −6.76) and with epicatechin (free energy −8.01). This enzyme could have important physiological implications in the metabolic change by readjusting the flow of ATP (adenosine triphosphate) production as it supplies NADH (nicotinamide adenine dinucleotide) to the mitochondrial respiratory chain [30], which will later be used for ATP synthesis [31,32]. In tumor cells, ATP rates are high, and it is usually proportional to the number of viable cells, as can be seen in the results presented, as a decrease of this nucleotide can be seen, which may be due to the interaction with this enzyme.

Interactions with insulin-like growth factor receptor 1 (IGF-1R) is shown, with −13.8, −14. 2, −2.37 and −3.44 free energy for ellagic acid, 3,4-dihydroxybenzoic acid, catechin and epicatechin, respectively. Phenolic compounds are potent inhibitors of this factor and can block one or more steps in the IGF-1R signaling pathway [33]. In cancer cells, IGF-1R plays a decisive role in contributing to the promotion of tumor growth by inhibiting apoptosis [34], transformation, metastasis [35] and the induction of angiogenesis in breast cancer through activation of S100A7 (the S100 calcium-binding protein A7)/RAGE (Receptor for Advanced Glycation End Products), which is associated with increased vascular endothelial growth factor (VEGF), so that inactivation of this factor would cause the inhibition of tumor cell growth [36].

**Figure 6:**
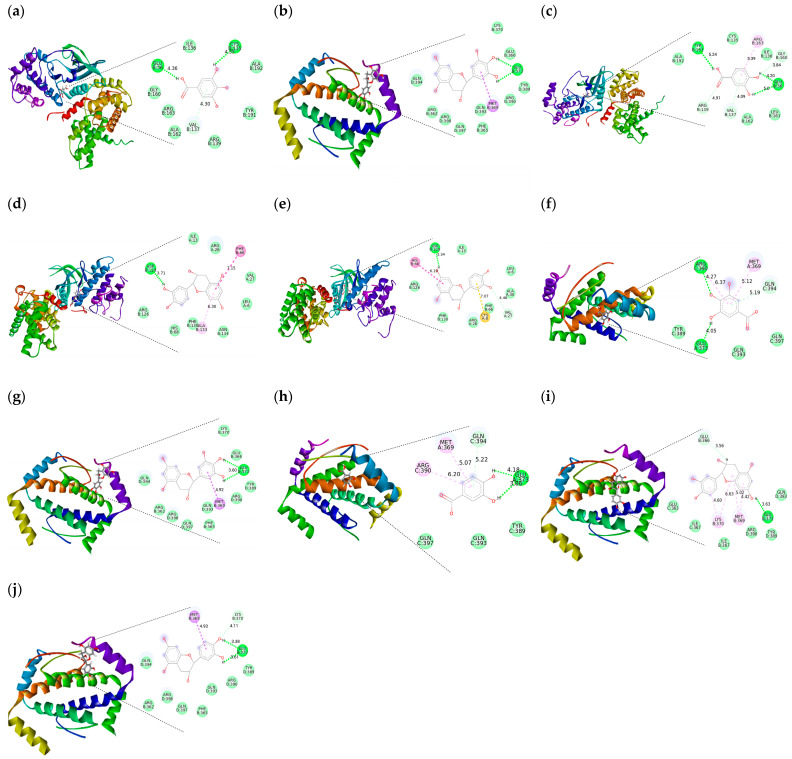
Interaction of CDK4 with (**a**) gallic acid, (**b**) epigallocatechin, (**c**) 3,4-dihydroxybenzoic acid, (**d**) catechin and (**e**) epicatechin. Interaction of p53 with (**f**) gallic acid, (**g**) epigallocatechin, (**h**) 3,4-dihydroxybenzoic acid, (**i**) catechin and (**j**) epicatechin. Amino acid residues in the compound–protein interaction are shown.

CDK1 interactions with ellagic acid, 3,4-dihydroxybenzoic acid, catechin and epicatechin present −14.84, −12.02, −3.53 and −5.35 free energy, respectively. CDK1 forms a complex with cyclin B, which is essential for the cell to progress from the G2 phase to mitosis [37]. CDK2 interactions with ellagic acid, 3,4-dihydroxybenzoic acid, catechin and epicatechin have −10.44, −11.59, −3.22 and −0.97 free energy, respectively. CDK2 is vital for the transition from the S phase to the G2 phase [38], and CDK4 has a free energy of −11.98 for gallic acid, hydrogen bonds CysB135 and GluB356 and polar interaction ValB137 (Figure 6a); −0. 31 free energy for epigallocatechin; hydrogen bonds AspB129 and LeuA6; polar interactions MetB369, CysA8 and HisB68 and hydrophobic interactions AlaB133 and ArgA26 (Figure 6b); 3,4-dihydroxybenzoic acid had −11.21 free energy; hydrogen bonds TyrB191, GluB56, ArgB139 and ValB137; polar interaction GlyB160 and hydrophobic interaction ArgB163 (Figure 6c); −7.51 free energy for catechin, hydrogen bond AspB129, polar interaction PheB66 and hydrophobic interaction AlaB133 (Figure 6d) and −1.51 free energy for epicatechin, hydrogen bonds AspB129 and ValA27 and polar interaction HisB68 (Figure 6e) (Supplementary Table S2). CDK4 is essential for the transition from the G1 phase to the S phase [39].

Among the proteins that stand out in cell cycle control is p53, with shows interactions with gallic acid, with a free energy of −15.29, hydrogen bonds ArgC390 and GluA373, polar interaction GlnC394 and hydrophobic interactions MetA369 and ArgC390 (Figure 6f); −0. 93 free energy for epigallocatechin, hydrogen bond GluB373 and polar interaction MetB369 (Figure 6g); for 3,4-dihydroxybenzoic acid, −14.98 free energy, hydrogen bonds ArgC390 and GluA373, polar interaction GlnC394 and hydrophobic interactions MetA369 and ArgC390 (Figure 6h); −6.79 free energy for catechin, hydrogen bond GluB373 and GluB366 and hydrophobic interaction MetB369 (Figure 6i) and −3.16 free energy for epicatechin, hydrogen bonds Glu and LysB370 and B373 and polar interaction MetB369 (Figure 6j). p53 can stop proliferation or induce apoptosis [29], and these interactions could explain the observed effect of reducing cell viability and/or invasion (Table 3).

TNFR1 showed interaction with ellagic acid and 3,4-dihydroxybenzoic acid, with a free energy of −9.45 in both cases, and did not interact with catechin and epicatechin. It is a protein involved in the promotion and control of inflammatory cytokines [40], which is a hallmark of inflammation-associated cancer [41]. TNFR1 activates some proteins such as RIPK1 (receptor-interacting serine/threonine-protein kinase 1) and RIPK3 (receptor-interacting protein kinase 3); these interactions promote the recruitment and phosphorylation of mixed lineage kinase domain-like protein (MLKL), which is the final executor of necroptosis [42]. MLKL had strong interaction with ellagic acid (free energy of −18.27) and 3,4-dihydroxybenzoic acid (free energy of −10.82) but no interaction with catechin and epicatechin (Table 3).

The metalloproteinases show interactions with ellagic acid, 3,4-dihydroxybenzoic acid, catechin and epicatechin, with free energy values of −17.47, −18.72, −3.35 and −3.74 for MMP2 and −9.52, −16.25, −4.67 and −3.96 for MMP9, respectively (Table 3). MMPs modify the cell structure by degrading components of the cell matrix and facilitate the invasion and metastasis of tumor cells [43,44]. E-cadherin is mostly expressed in normal cells, since it is in charge of cell–cell junctions; on the other hand, N-cadherin is in greater quantity in tumor cells facilitating motility and invasion during tumor progression [45,46].

### 2.4. Three-Dimensional Cell Model Assays

#### 2.4.1. EAB Decreases Cell Viability in the 3D Model by Assessing Plasma Membrane Integrity in MCF-7 and MDA-MB-231 Cells

Three-dimensional cell cultures allow the creation of more relevant tumor models that facilitate the investigation of neoplastic cell progression and responses to treatment [47].

**Figure 7:**
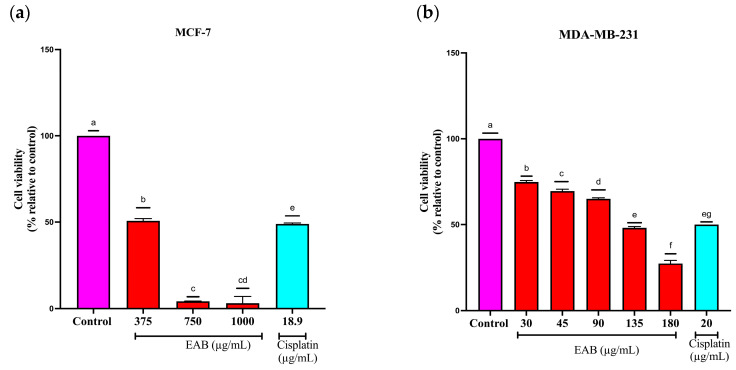
Effect of EAB on the cell viability of breast cancer cells in the 3D model: (**a**) MCF-7; (**b**) MDA-MB-231. Results are expressed as mean ± SD values. *p* < 0.0001 according to Tukey’s post hoc test, *n* = 6. Different letters represent significant differences between types of treatment.

As can be seen in Figure 7, the MCF-7 cell line showed a decrease in cell viability of 50 and 95% for concentrations 375 and 750 µg/mL concerning the control and of 25, 30, 35, 50 and 75% for concentrations 30, 45, 90, 135 and 180 µg/mL in the MDA-MB-231 cell line, which indicates that this cell line is more sensitive to these treatments, although there is a difference in comparison with 2D cell viability, which is attributed to the three-dimensional structure of the spheroid, and the final concentration of the treatment in the deeper layers is so low that it is ineffective against the cells [48], which happens in conditions of malignant neoplasms; however, the minimum inhibitory concentration of EAB could suggest that it is a good alternative.

#### 2.4.2. EAB Decreases ATP Levels of MCF-7 and MDA-MB-231 Cells in the 3D Model

ATP is a fundamental nucleotide for obtaining cellular energy [49]. One of the characteristics found in carcinogenesis is the presence of high levels of ATP, a phenomenon also known as Warburg, where cancer cells use glycolysis to generate lactate, even under conditions of cellular normoxia [50].

Since the amount of ATP is directly proportional to the number of viable cells, and considering that, in the tumor environment, there is metabolic dysregulation, one of the best-known characteristics of cancer is the assessment of cell viability by quantifying ATP levels and is one of the most accurate methods.

**Figure 8:**
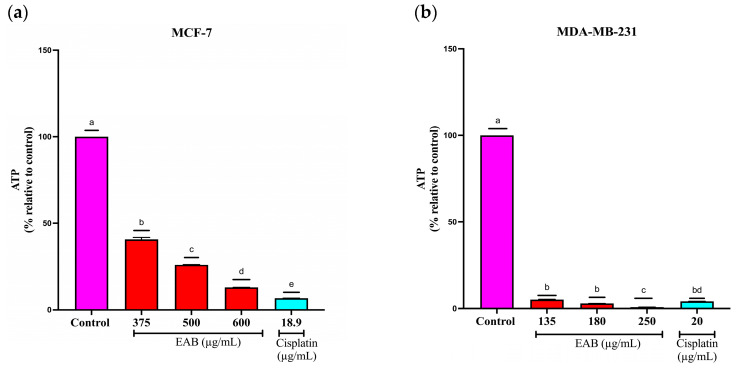
ATP level decrease in the 3D model of breast cancer cells with EAB treatment: (**a**) MCF-7; (**b**) MDA-MB-231. Cell viability determined by ATP level quantification with a CellTiter-Glo^®^ 3D kit (PROMEGA). Results are expressed as mean ± SD values. *p* < 0.0001 according to Tukey’s post hoc test, *n* = 6. Different letters represent significant differences between types of treatment.

For the MCF-7 cell line, concentrations 375, 500 and 600 µg/mL were used, observing a significant decrease in the ATP levels after 24 h, decreasing cell viability by 59, 74 and 87%, respectively, while cisplatin decreased it by 93%. On the other hand, the concentrations used for the MDA-MB-231 cell line were 135, 180 and 250 µg/mL, decreasing cell viability 95, 97 and 99%, respectively, while cisplatin in this cell line decreased viability 96% (Figure 8), showing a high sensitivity to the extract compounds.

#### 2.4.3. EAB Activates Caspases-3/7, -8 and -9 in MCF-7 and MDA-MB-231 Cell Lines in a 3D Model

Apoptosis is an evolutionarily conserved programmed cell death process that is essential for normal tissue homeostasis, participating in the elimination of potentially dangerous cells, including tumor cell precursors [51]. One of the key elements in the regulation of apoptosis is constituted by caspases. Caspases are synthesized as inactive precursors that are converted to the active form by proteolytic cleavage. Thus, the initial activation of one caspase triggers a chain reaction leading to the activation of other caspases and cell death either by an intrinsic or extrinsic pathway [52]. Therefore, the regulation of caspase activation is critical in determining cell survival. Depending on the role they play in the apoptotic process, they are divided into initiator caspases-1, -2, -4, -5, -8, -9, -10, -11 and -12, which present pro-domains that allow their binding to signaling complexes and executioner caspases-3, -6, -7 and -14, which are responsible for triggering apoptosis [53].

To understand the mechanism of regulation at the caspases level, as well as the pathway by which the apoptotic process is triggered, the activities of caspases-3/7, -8 and -9 were evaluated.

**Figure 9:**
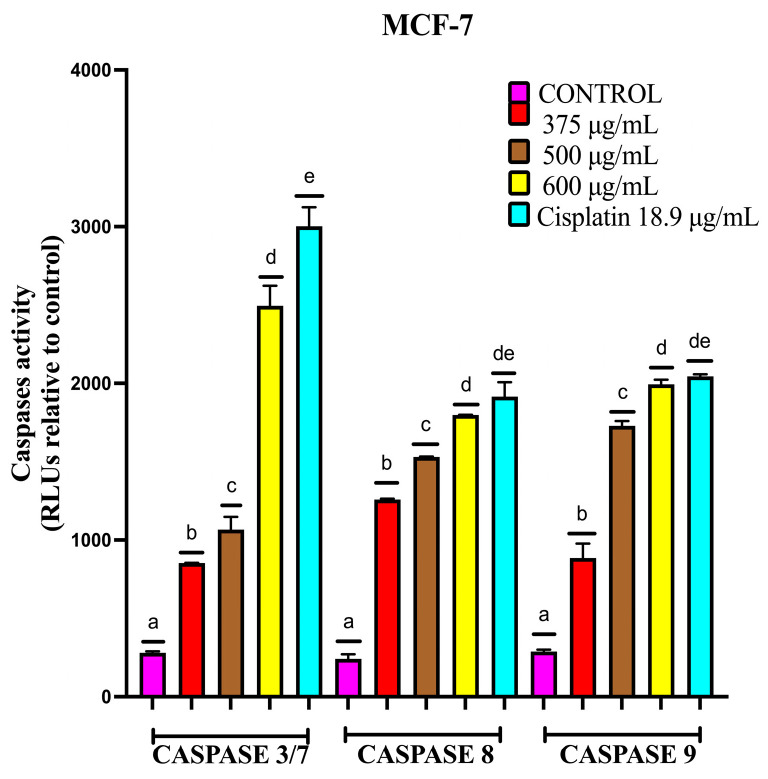
Activity of apoptosis initiator and effector caspases in the 3D model of MCF-7 cells. Induced apoptosis as determined by the activity of caspases-3/7, -8 and -9. Spheroids exposed to 375, 500 and 600 μg/mL EAB and 18.9 μg/mL cisplatin for 24 h. Mean values and standard deviation of relative fluorescence units by Tukey’s test of a posteriori comparisons (*p* ≤ 0.0001), *n* = 12. Different letters represent significant differences between treatment types.

As shown in Figure 9 in the MCF-7 line, after treatment with EAB at 375, 500 and 600 µg/mL and 18.9 µg/mL of cisplatin after 24 h of exposure in the spheroids, an increase in the activity of caspases-3/7, -8 and -9 was observed, with a notable increase in the activity of caspase-3/7 and, in the case of caspases-8 and -9, shows increased activity, and at the highest concentration used, there is no significant difference compared to cisplatin, indicating that both the extrinsic and intrinsic pathways are activated. In all cases, the greatest increase in caspase activity occurred at 600 µg/mL.

**Figure 10:**
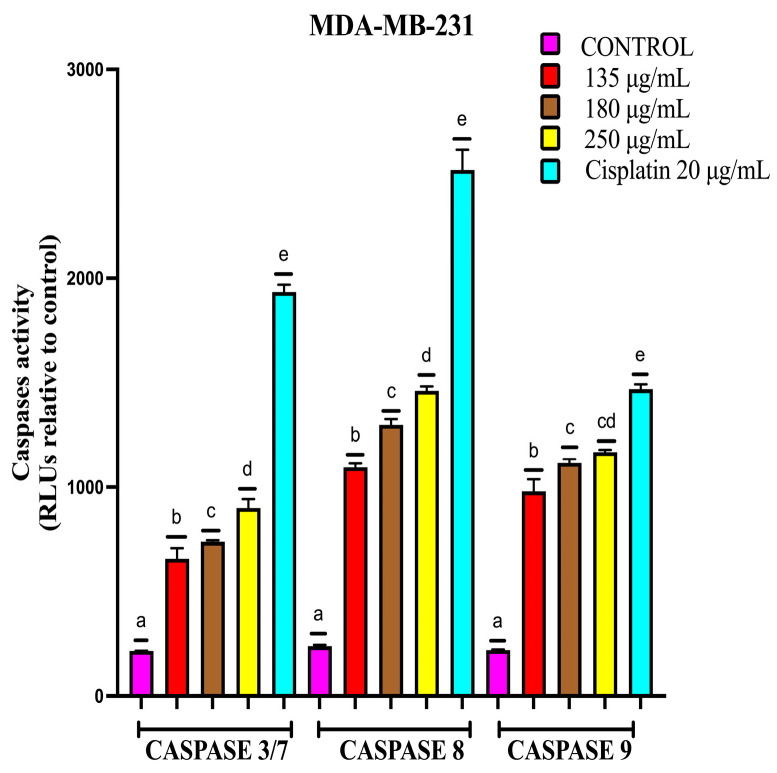
Activity of apoptosis initiating and effector caspases in the 3D model of MDA-MB-231 cells. Spheroids exposed to 135, 180 and 250 μg/mL EAB and 20 μg/mL cisplatin for 24 h. Mean values and standard deviation of relative fluorescence units by Tukey’s test of a posteriori comparisons (*p* ≤ 0.0001), *n* = 12. Different letters represent significant differences between treatment types.

The evaluation of caspase activity in MDA-MB-231 showed an increase in the activity of caspases-3/7, -8 and -9, although the levels were lower compared to cisplatin (Figure 10). Comparing the effect of the extract on the different cell lines, the activity of caspases-3/7 and -9 increased more in MCF-7 than in MDA-MB-231, and caspase-8 showed a higher increase in the MDA-MB-231 cell line.

#### 2.4.4. Inhibition of Cell Invasion by EAB in MCF-7 and MDA-MB-231 Cells in a 3D Model

Metastasis is one of the characteristic processes of malignant tumors [54] and is one of the hallmarks of cancer [55]. Tumors undergo a process known as epithelial–mesenchymal transition (EMT) to evade the immune system to perform invasion and colonization of adjacent and distant tissues.

**Figure 11:**
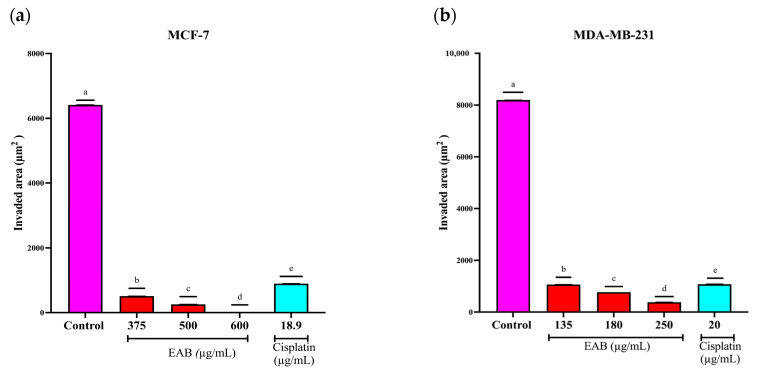

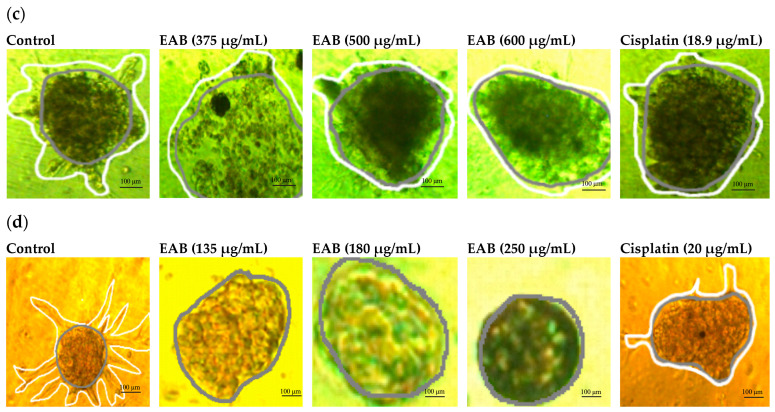
Inhibition of tumor invasion in a 3D model of MCF-7 and MDA-MB-231 cells. Invasion determined by area and perimeter of the invaded Matrigel. Mean values and standard deviation of relative fluorescence units by Tukey’s test of a posteriori comparisons (*p* ≤ 0.0001), *n* = 6. (**a**) Invaded area (μm^2^) in MCF-7 cells. Different letters represent significant differences between treatment types. (**b**) Invaded area (μm^2^) in MDA-MB-231 cells. Different letters represent significant differences between treatment types. (**c**) Spheroid images of MCF-7 cells taken under inverted microscopy with a scale of 100 μm and a magnification of 20×. The gray line represents the delimitation of the spheroid, while the white line represents the border of the invading cells. The invaded area is the area between the gray and white lines. (**d**) Images of spheroids of MDA-MB-231 cells taken under inverted microscopy with a scale of 100 μm and a magnification of 20×. Gray-colored line represents the delimitation of the spheroid, while white-colored line represents the border of the invading cells. The invaded area is the area between the gray line and the white line.

As mentioned above, the inhibition of tumor invasion is of utmost importance. In this study, cell invasion was determined by measuring the invaded area of the Matrigel. As shown in Figure 11, the invaded area decreased significantly after exposing MCF-7 and MDA-MB-231 cell spheroids for 24 h with EAB at different concentrations, and the invaded area was even smaller than with cisplatin.

#### 2.4.5. EAB in Combination with Cisplatin and Paclitaxel Decreases Viability of MCF-7 and MDA-MB-231 in the 2D and 3D Models

The low specificity of the current treatments for cancer is reflected in the rapid generation of drug resistance and the deterioration of the quality of life of patients suffering from this disease [56]. Cisplatin, one of the most widely used drugs in chemotherapy, has two main limitations: its adverse effects, such as nephrotoxicity, nausea, vomiting, ototoxicity, peripheral neuropathy and myelosuppression, and intrinsic or acquired resistance, increasing the dose in patients and thus increasing toxicity [57].

As shown in Table 4, in the 2D model, the effect of IC_50_ cisplatin in combination with IC_50_EAB was enhanced, and compared to their IC50s, an inhibition of 75% was achieved for the MCF-7 cell line and 69% for the MDA-MB-231 cell line. In the 3D model (Table 5), it is shown that the effect of the combination of the ${IC}_{50}$ of the antineoplastic and the extract inhibit cell viability by 75% in MCF-7 and 72% for MDA-MB-231.

The combination of paclitaxel and phenolic compounds is a field of growing interest due to the effect they have when combined, improving therapeutic efficacy and mitigating side effects such as peripheral neuropathy, which is one of the most prevalent and debilitating of paclitaxel and affects approximately 60–97% of patients undergoing this treatment [58,59].

As shown in Table 4, the combination of IC_50_ EAB + IC_50_ paclitaxel increased the inhibition in the cancer lines used, being 86% in MCF-7 and 75% in MDA-MB-231 in the 2D model, and in the 3D model, there was also an increase in cell inhibition, being 76% for the MCF-7 cell line and 70% for the MDA-MB-231 cell line, as shown in Table 5, compared to when used individually.

## 3. Discussion

Research on anticancer phytochemicals has increased due to reduced or no adverse effects on normal cells [60]. The unique composition profiles of the compounds present in each genus and plant species produce combinations that have different effects in each case, known to have promising biological effects, such as antioxidant and cytotoxic potential. The results of this chemical profile are significantly better than those found in other plants, and this is particularly notable given the scarcity of 3D model studies. The results from the 2D model showed that EAB is cytotoxic in the MCF-7 and MDA-MB-231 cell lines. On the other hand, in the clonogenic assay, no colony formation was observed, a relevant result because, when a tumor resists treatment, not all neoplastic cells are eliminated, which can promote patient relapse. Among the mechanisms that promote drug resistance are decreased drug uptake, activation of DNA (deoxyribonucleic acid) repair mechanisms and inhibition of apoptosis [61,62,63]. Some membrane transport proteins are known to be involved in drug resistance by altering drug transport and pumping drugs out of tumor cells [61,64,65], such as ABCB1 (ATP-Binding Cassette Subfamily B Member 1), which expression determines a tumor phenotype resistant to several drugs, such as vinca alkaloids, epipodophyllotoxins, taxanes and anthracyclines, and ABCG2 (ATP-Binding Cassette Subfamily G Member 2 (JR Blood Group)), a protein associated with breast cancer resistance [66]. Overexpression of these proteins driven by transcription factors, such as HIF-2α (hypoxia-inducible factor 2α), can lead to increased antineoplastic efflux and resistance to antineoplastic drugs [64]. Therefore, preventing cells from proliferating becomes vitally important, and this can be achieved through the cell cycle. A cell undergoes a series of events in which it duplicates its genome, grows and divides into two cells. This process consists of four stages: G1, S, G2 and M [67]. At strategic points in the cell cycle, signaling pathways monitor the completion of specific events before moving on to the next stage, a process regulated by pathways known as checkpoints. These pathways include sensor proteins that detect these lesions and simultaneously trigger two processes: They recruit additional effector complexes to correct the problems and activate signaling pathways that induce a temporary halt in the cell cycle [68]. In cancer cells, this process does not occur, and the cycle progresses due to mutations in the proteins involved, making it essential to arrest the cell cycle of these cells.

Positive regulation and progression in the cell cycle are carried out through the interaction of two proteins: cyclins and cyclin-dependent kinases. Only one set of CDK–cyclin complexes participates in cell cycle progression, including CDK1 (cyclin-dependent kinase 1), CDK2 (cyclin-dependent kinase 2), CDK4 (cyclin-dependent kinase 4) and CDK6 (cyclin-dependent kinase 6), while 10 cyclins belong to four classes (A, B, D and E) [69]. As shown in Figure 4a, the CDK–cyclin complexes involved in the G1 phase of the cell cycle are D-CDK4/6; this is important, because the extract arrested the cell cycle in this phase. Therefore, the interaction of EAB with the D-CDK4 complex is observed. When cyclin is inhibited, the complex becomes inactive and cannot phosphorylate the retinoblastoma (Rb) tumor suppressor protein, which does not release the E2F transcription factor and therefore cannot stimulate the transcription of CDK2 and cyclin E necessary for the transition to the S phase of the cell cycle (Figure 4b) [28].

Chen et al. [70] conducted a study with gallic acid in combination with hesperidin, demonstrating that it causes colorectal cells to arrest in the G1 phase, resulting in increased levels of p21, a protein that inhibits the CDK4/6 complex and thus stops cell division. The importance of arrest in this phase lies in the fact that cells evaluate their environment and prepare for DNA replication, which must be carried out under optimal conditions. It is crucial, because it acts as a control mechanism, determining whether a cell divides or enters a resting state [71]. This phase is essential for cell differentiation. If the cell passes this checkpoint, the cell cycle will progress regardless of external signals; however, passing it requires that many previously completed tasks have been accomplished and the assistance of external stimuli.

However, as shown in Table 2, some cancer cells can overcome the G1 checkpoint and progress to the S and G2 phases despite CDK4/6 inhibition, as shown in the work of Pandey et al. [72], in which the CDK4/6 inhibitor Palbociclib was combined with Eribulin to destroy cells that escaped to the following phases of the cell cycle. Tian et al. [73] hypothesized that the RhoA pathway (member A of the Ras homologous family) plays an active role in promoting cell migration in the G1 phase, as it controls mitogenic pathways such as ERK (extracellular signal-regulated kinases) and activates proteins such as Par6 (partitioning defective protein 6), which is key in cell division, and modulates the expression of cell cycle regulatory molecules such as cyclin D1 [74]. There may be an increase in the expression of the p53 protein, which can lead to interactions with members of the Bcl-2 family, which regulate the process of apoptosis by interacting with the outer mitochondrial membrane to release cytochrome C and initiate the apoptotic cascade [75]. The compounds present in this extract are known to increase the activity of this protein, as is the case with 3,4-dihydroxybenzoic acid [76] and gallic acid, which increase the p53 levels and promote apoptosis in breast cancer [77,78].

As in the 2D model, a decrease in cell viability is observed in the 3D model, with IC_50_ values of 375 µg/mL for the MCF-7 cell line and 135 µg/mL for MDA-MB-231. This type of model is one of the most widely used today, as it is more similar to the expected behavior of the tumor environment, providing a more accurate picture of the effect of treatments on cell lines [79]. As can be seen, there is a 7-fold increase in concentration for the MCF-7 cell line and a 30-fold increase for the MDA-MB-231 cell line. In 3D in vitro models, an increase in the concentration of compounds is generally required compared to 2D models to achieve the same response. This varies depending on the compound or cell line, with reported increases in concentrations ranging by 6–30 times [80].

The disparity in sensitivity between 2D and 3D models in cell cultures is well known. A549 lung cancer cells exhibit greater resistance to doxorubicin in 3D cultures compared to a 2D model [81]. Applying both models, there is greater resistance to the drug in the three-dimensional model compared to the two-dimensional model [82]. This is because, in these models, three-dimensional masses are formed with an architecture that allows for the presence of an extracellular matrix and cell layers, which influences the availability of compounds to cells, variable access to nutrients and exposure to treatments. Therefore, the response of the 3D model to the compounds is different from that of the two-dimensional models. Another method of cell viability to corroborate the cytotoxic effect of EAB is to measure the ATP levels. ATP is a nucleotide essential for cellular energy production [49]. One of the characteristics of carcinogenesis is the presence of high levels of ATP, a phenomenon also known as the Warburg effect, where cancer cells use glycolysis to generate lactate, even under conditions of cellular normoxia [50].

Given that the amount of ATP is directly proportional to the number of viable cells, and considering that metabolic dysregulation is one of the best-known characteristics of cancer in the tumor environment, evaluating cell viability by quantifying the ATP levels is one of the most accurate methods.

As can be seen, treatment with EAB increased the inducer and effector caspases. This could be due to the compounds present in this extract, such as ellagic acid, which increase the levels of the Fas/FasL death receptor protein, which are the initiators of the extrinsic apoptosis cascade that activates caspase-8 [83], and quercetin, which increases the expression of DR5 (TRAIL receptor) through stress-mediated mechanisms, facilitating the activation of TRAIL receptors [84] and promoting the recruitment of FADD and the recruitment of procaspase 8 [85], leading to the formation of the DISC complex. DISC activates caspase-8, which, in turn, directly activates effector caspases-3 and -7 [86].

This is relevant for the MDA-MB-231 cell line, which is more sensitive to this apoptotic pathway [87]. From above, we can conclude that the increase in the activity of inducer and effector caspases shows the induction of apoptosis in both cell lines and that the activity is dose-dependent.

During the EMT process, cell–cell and cell–basement membrane interactions are lost, and cells lose their shape due to a rearrangement of the actin cytoskeleton and the loss of polarity typical of the epithelial phenotype through genotypic changes [88]. These cells acquire a mesenchymal phenotype characterized by high invasive capacity and resistance to apoptosis [89]. It is responsible for 90% of deaths in cancer patients and represents the greatest challenge for oncology [90].

This process is characterized by invasion of the extracellular matrix, penetration of blood and/or lymphatic vessels, dissemination through the circulatory system, cell blockage at the level of the capillaries of the target organs, extravasation of tumor cells, infiltration of the surrounding parenchyma and evasion of the host’s defenses [91]. This can be attributed to the fact that the in silico study carried out in this work observes the interaction of some compounds, such as gallic acid and 3,4-dihydroxybenzoic acid, with N-cadherin, a molecule closely related to invasion and metastasis, as it is a mesenchymal marker in which cells transform from an immobile epithelial phenotype to a migratory mesenchymal phenotype. Therefore, the positive regulation of N-cadherin increases the ability of tumor cells to invade and metastasize to different sites, in addition to the fact that this molecule increases the transcriptional activity of β-catenin 27-fold [92], which simultaneously increases the expression of genes encoding proteins such as MMP-9 metalloproteinase [93], which main function is to degrade components of the cellular matrix [94]. As mentioned above, the results suggest that EAB is downregulating this molecule, leading to a decrease in cell invasion.

The combination of cisplatin and phenolic compounds has been increasingly recognized for its proven anticancer efficacy and ability to mitigate adverse effects. Recent studies have demonstrated that several phenolic compounds can sensitize cancer cells to cisplatin, thereby enhancing its efficacy and reducing its toxicity [95]. In addition to enhancing the anticancer effects of cisplatin, phenolic compounds also exhibit protective properties against cisplatin-induced toxicity by reducing the oxidative stress and inflammation associated with its treatment, thus protecting renal function and maintaining the anticancer efficacy of the drug [96]. Certain phenolic compounds can inhibit organic cation transporters involved in the renal absorption of cisplatin, which could reduce its nephrotoxic effects while actively acting against tumors [97]. According to the Chou-Talalay [98] method, the combination of cisplatin and EAB produced a combination index less than 1 in cells, and this indicates that there is a synergistic interaction, since the combination was more effective than the individual compounds. This may be because the extract contains gallic acid, 3,4-dihydroxybenzoic acid, catechin, epicatechin and ellagic acid, which can enhance the effect of cisplatin on apoptosis induction through the JAK/STAT3 pathway, leading to an increase in pro-apoptotic factors [99] and counteracting drug resistance.

Coadministration of phenolic compounds with paclitaxel can significantly improve tumor suppression [100]. Aborehab et al. [77] demonstrated the synergistic effect between ellagic acid and paclitaxel in reducing the proliferation of a breast cancer cell line (MCF-7). Similarly, with the anticancer agent cisplatin, the combination index with paclitaxel was less than 1.

This effect may be attributed to the inhibition of the mitotic cycle; increased apoptosis and overexpression of p53, Bax and caspase-3 in these cells. Furthermore, numerous studies have demonstrated the synergistic effect between catechins and paclitaxel [101]. Luo et al. [102] showed that treatments with epigallocatechin gallate in combination with paclitaxel increased apoptosis in a mouse model of breast cancer compared to the use of either anticancer agent alone, thanks to the activation of JNK (Jun N-terminal kinases). JNKs, in turn, activate apoptotic signaling either by regulating pro-apoptotic genes, transactivating specific transcription factors such as c-Jun, or directly modulating the activities of pro-apoptotic mitochondrial proteins [103].

This suggests that the incorporation of phenolic compounds into treatments could improve outcomes in patients with aggressive cancers who are often resistant to standard therapies. This dual action suggests its potential as a safer and more effective adjuvant therapy in cancer treatment, helping to reduce the side effects caused by first-line antineoplastic drugs.

On the other hand, studies in animal models are necessary to determine whether the behavior of these combinations in an environment where cell signaling pathways interact is the same as that observed in cancer cell lines and to finally apply translational medicine.

## 4. Materials and Methods

### 4.1. Cell Culture

The MCF-7 (ATCC^®^ HTB-22^™^) and MDA-MB-231 (ATCC^®^ HTB-26^™^) cell lines were grown in DMEM GIBCO (Thermo Fisher Scientific Inc., Waltham, MA, USA) and Leibovitz-L15 medium, respectively. Both were supplemented with 10% GIBCOTM fetal bovine serum (FBS) (Thermo Fisher Scientific, Waltham, MA, USA) and 1% antibiotic-antimycotic ${GIBCO}^{TM}$ (Thermo Fisher Scientific). Cultures were maintained under aseptic conditions at 37 °C and 5% ${CO}_{2}$.

### 4.2. Preparation of Extracts

The ground plant (1 g) was dissolved in 5 mL of ethyl acetate, shaken for 30 min and then filtered through filter paper (Whatman No. 1). The supernatant was volumetrically adjusted to the initial volume, which was designated as *Ballota hirsuta* Benth ethyl acetate extract (EAB). For subsequent analysis in the cells, all the solvent was evaporated, then resuspended in 0.1% DMSO and in a medium.

### 4.3. Ultra-Performance Liquid Chromatography with Time-of-Flight Mass Spectrometry (UPLC-Q-TOF/MS^E^) and Quantification of Compounds in Crude Extract by HPLC

EAB was injected into the Ultra-Performance Liquid Chromatograph with Time-of-Flight Mass Spectrometry (UPLC-Q-TOF/MS Xevo) (model G2-XS, Waters, Milford, MA, USA). All separations were carried out using a Waters ACQUITY UHPLC^®^ HSS T3 (2.1 mm × 100 mm, 1.8 μm particle size) at a column temperature of 40 °C and an autosampler temperature of 7 °C. The sample volume was 5 μL each time, and the liquid flow rate was 0.45 mL/min. The ionization parameters were as follows: the cone voltage was 15 V, and the capillary voltage was 2.5 kV in the negative mode. The desolvation temperature was set at 550 °C, while the temperature of the ion source was maintained at 120 °C. The desolvation gas (N_2_) flowed at 1000 L/h, while the cone gas (N_2_) flowed at 50 L/h. Data acquisition was performed using MassLynx software (V4.1. Waters Corporation, Milford, MA, USA). The UNIFI 1.8.0 platform (Waters, Manchester, UK) was used to analyze the metabolites in the samples. The unique mass spectrometry fragment patterns observed were compared with fragment ions documented in the literature and data libraries such as PubChem (https://pubchem.ncbi.nlm.nih.gov/, accesed on 10 April 2025), Massbank (https://massbank.eu/MassBank/, accesed on 10 April 2025) and ChemSpider (https://www.chemspider.com/, accesed on 10 April 2025) through the final identification of the metabolites [104,105,106]. A HPLC-UV analysis was carried out for the determination of the different compounds present in the ethyl acetate extract of *Ballota hirsuta* Benth, and said determination was carried out by comparison with a standard curve of an authentic sample of each standard. The concentrations used were 200, 150, 100, 75, 50 and 25 µg/mL; 1200, a Zorbax C18 analytical column of 25 × 4.6 cm, was used as the stationary phase and acetonitrile and water as the mobile phase. Then, 10 µL of the samples were injected at a flow rate of 1 mL/min. The detection of analytes was carried out at 250, 254 and 280 nm.

### 4.4. Culture

The cultures at 90% confluence were treated with a 0.025% EDTA-trypsin solution for 2 min at 37 °C. An aliquot was taken from the suspension obtained by trypsinization and then stained with 4% trypan blue, and the cells were counted in a Neubauer chamber.

### 4.5. Two-Dimensional Cell Model Assays

#### 4.5.1. Two-Dimensional Cell Viability by SRB (Sulforhodamine B)

First, 20,000 MCF-7 and MDA-MB-231 cells per well were seeded into a 96-well plate in 90 µL of medium. These were treated with EAB after removal of the solvent from extraction and dissolved in DMSO (0.1%) and the medium at different concentrations: 25, 50, 75, 100, 125, 150, 175, 175, 200, 251, 502, 753 and 1004 µg/mL for MCF-7 and 2, 3, 4, 5, 6, 7,8, 12, 17, 22, 220, 440, 660 and 806 µg/mL for the MDA-MB-231 cell line and incubated for 24 h. Without removing the cell culture, 100 µL of cold 10% TCA (trichloroacetic acid) was carefully added to each well, and the plate was incubated at 4 °C for 1 h. The plates were washed 4 times with distilled water, and the excess was removed with absorbent paper towels.

Then, 100 µL of 0.057% SRB (Sulforhodamine B) solution was added to each well and left at room temperature for 30 min. Then, the plates were quickly washed four times with 1% acetic acid to remove any unbound dye, 200 µL of 10 mM Tris solution was added to each well and the plate was shaken for 5 min to solubilize the protein-bound dye. Measurement was performed at 510 nm on a plate reader (SYNERGY H1 BioTek, Winooski, VT, USA) [107].

#### 4.5.2. Clonogenic Assay in a 2D Culture of MCF-7 and MDA-MB-231 Cells

The effect of EAB on cell colony formation was evaluated by clonogenic assay. First, 500 MCF-7 and MDA-MB-231 cells were seeded in 6-well plates in growth medium. Subsequently, the cells were exposed to concentrations of 50 and 100 μg/mL of the extract and incubated for 72 h. The medium was then removed and replaced with fresh growth medium, and the culture was incubated for nine days. After colony formation, the medium was removed, and the cells were washed with cold PBS. The colonies were then fixed with cold 4% formaldehyde for 30 min. The cells were stained with 0.5% crystal violet in 96% ethanol for 15 min. Finally, the cells were washed and dried overnight, and the number of colonies was counted using ImageJ Software version 1.54g [108].

#### 4.5.3. Cell Cycle Assay in a 2D Model in MCF-7 and MDA-MB-231 Cells by Flow Cytometry

This assay was performed according to Gomes et al. [109], with some modifications. First, 200,000 MCF-7 and MDA-MB-231 cells were seeded in a 3.5 cm 6-well plate (Nest^®^, Palo Alto, CA, USA) (200,000/mL) and then stimulated to synchronize in G0 with serum-free culture medium for 24 h. Next, to bring the cells out of the G0 phase, the culture medium supplemented with 10% FBS was added in the presence of 500 and 1000 μg/mL EAB and 13 μg/mL cisplatin. After 24 h, cells were harvested, washed, centrifuged and fixed in 4% paraformaldehyde for 60 min. Subsequently, light-protected RnaseA and propidium iodide were added. The DNA content was analyzed using a CytoFLEX S flow cytometer (Beckman Coulter, Brea, CA, USA). A total of 20,000 events were acquired. FlowJo^TM^ Analysis Software version 10.10 was used for data analysis. The data presented are representative of those obtained in three independent experiments performed in triplicate.

#### 4.5.4. Cyclin-Dependent Kinase 4 (CDK4) Activity Inhibition Assay

The inhibition of CDK4 activity was determined with the CDK4^®^ kit (BPS Bioscience, San Diego, CA, USA), following the manufacturer’s specifications. For the inhibition assay, concentrations of 3.7, 49.3 and 100 μg/mL of EAB were used for 1 h. After the reaction time was over, 50 μL of Kinase-Glo^®^ was added to each well and incubated for 15 min more, and then, the luminescence was quantified with a microplate reader (SYNERGY H1, BioTek, Winooski, VT, USA).

#### 4.5.5. p53 Protein Transcription Assay

The p53 protein transcription assay was determined with the p53 Transcription Factor Assay Kit (Cayman Chemical, Ann Arbor, MI, USA) following the manufacturer’s specifications. The concentrations of 49.3 μg/mL for MCF-7 and 3.7 μg/mL of EAB for MDA-MB-231 were used for the assay, and absorbance was measured on a microplate reader (SYNERGY H1, BioTek, Winooski, VT, USA) at 450 nm.

### 4.6. In Silico Analysis

The interaction was carried out by molecular docking using Docking Server Software (http://swissdock.ch/, accessed on 13 April 2025) with the Attracting Cavities 2.0 (AC) algorithm composed of a CHARMM force field and fast analytical continuous treatment of solvation (FACTS) model. The sampling completeness was 90, but the cavity was prioritized at 90. During the search, a non-rotated 3D space was applied, a value of 0.2 Å for each step. The box sizes were determined as 20–20–20 to 25–25–25 with different box centers.

The proteins were selected from the Protein Data Bank (PDB): aldehyde dehydrogenase 1AE (PDB code 6TGW), IGF-R1 (PDB code 1K3A), CDK1 (PDB code 4YC3), CDK2 (PDB code 1HCL), CDK4 (PDB code 2w96), p53 (PDB code 2wq1), TNF-R1 (PDB code 2ZJC), MLKL (PDB code 4M67), MMP2 (PDB code 1CK7), MMP9 (PDB code 1L6T), E-cacherin (1Q1P) and N-Cadherin (PDB code 1NCG).

The structures of gallic acid, catechin, epicatechin, procyadinin b2, epigallocatechin gallate, gallocatechin 3-gallate, ellagic acid, epicatechin-3-O-gallate, quercitrin, phloridzin, 3,4-dihydroxybenzoic acid, gallic acid-4-O-(6-galloyglucoside), gallicacid-4-O-(6-galloyglucoside) and quercetin-3-O-glucoside were downloaded from PubChem (https://pubchem.ncbi.nlm.nih.gov/, accessed on 13 April 2025)and refined by UCSF Chimera version 1.17.2, a bioinformatics software program, eliminating solvent impurities. The simulation in BIOVIA Discovery Studio version 21.1.0.20298 uses the molecular dynamics (MD) method in the modeling of interactions between particles, calculating the strength of the positions of the molecules with the proteins.

### 4.7. Three-Dimensional Cell Model Assays

#### 4.7.1. Cell Viability by Plasma Membrane Integrity in a 3D Culture of MCF-7 and MDA-MB-231 Cells

First, 1200 cells of the MCF-7 and MDA-MB-231 lines were seeded in 96-well ultra-low-adherent plates (Corning^®^, Corning, NY, USA). After spheroid formation, the cells were exposed for 24 h to 375, 750 and 1500 μg/mL of EAB and 18.9 μg/mL of cisplatin for the MCF-7 cell line, while 30, 45, 90, 135 and 180 μg/mL of the same extract and 20 μg/mL of cisplatin were used for the MDA-MB-231 cell line. After treatment, Sytox Green^®^ dye was added, and the fluorescence was read on a microplate reader (SYNERGY H1, BioTek, Winooski, VT, USA).

#### 4.7.2. Quantification of ATP Levels in the 3D Model

The effect on cell viability was determined using the CellTiter-Glo^®^ 3D luminescent cell viability assay (PROMEGA, Madison, WI, USA) following the manufacturer’s specifications. First, 300 MCF-7 and MDA-MB-231 cells were seeded per well, and the spheroids were exposed to 375, 500 and 600 μg/mL EAB and 18.9 μg/mL cisplatin and 135, 180 and 250 μg/mL EAB and 20 μg/mL cisplatin, respectively, for 24 h. CellTiter-Glo^®^ reagent was added, incubated for 30 min, and then, the luminescence was quantified in a microplate reader (SYNERGY H1, BioTek, Winooski, VT, USA).

#### 4.7.3. Caspases-3/7, -8 and -9 Activity of MCF-7 and MDA-MB-231 Cells in the 3D Model

Caspase activity was determined with the Caspase-Glo^®^ kit for caspases-3/7, -8 and -9 (Promega Corp., Madison, WI, USA) following the manufacturer’s specifications. First, 300 MCF-7 cells were seeded per well, and the spheroids were exposed to 375, 500 and 600 μg/mL EAB and 18.9 μg/mL cisplatin, and for the MDA-MB-231 cell line, 135, 180 and 250 μg/mL EAB and 20 μg/mL cisplatin were used for 24 h; at the end of the incubation period, the corresponding Caspase-Glo^®^ reagent was added. Subsequently, it was incubated for 1 h, and then, the luminescence was quantified using a microplate reader (SYNERGY H1, BioTek, Winooski, VT, USA).

#### 4.7.4. Tumor Invasion Assay in a 3D Model of MCF-7 and MDA-MB-231 Cells

The invasion assay was performed as described by Martinez-Rodriguez et al. [110], with some modifications. First, 1200 MCF-7 and MDA-MB-231 cells were seeded per well, and after spheroid formation, the MCF-7 cells were treated with 375, 500 and 600 of EAB and 18.9 of cisplatin, and the MDA-MB-231 cells with 135, 180 and 250 μg/mL of EAB and 20 μg/mL of cisplatin for 24 h. Subsequently, 100 μL of Matrigel^®^ Matrix (Corning^®^) per well and then DMEM (MCF-7) and Leibovitz-L15 (MDA-MB-231) were added. Invasion was determined using a Zeiss Axiovert 25 inverted microscope (Carl Zeiss, Oberkochen, Germany) and ImageJ software version 1.54g, where the area of invasion was calculated by measuring the perimeter between the edge of the spheroids and the edge of the invading cells.

### 4.8. Statistical Analysis

Data are expressed as means. Results were analyzed by one-way ANOVA using Graph Prism software version 9.4.0, mean values and standard deviation of the relative units of fluorescence and absorbance by Turkey’s test of a posteriori comparisons *(p* < 0.0001). Different letters represent significant differences between treatment types. For the analysis of flow cytometry data, FlowJo^TM^ software version 10.10 was used, and the data presented were representative of those obtained in three independent experiments performed in triplicate.

## 5. Conclusions

The results presented in this study suggest that Ballota hirsuta Benth extract has therapeutic potential in aiding cancer treatments by significantly reducing cell viability in 2D and 3D models of MCF-7 and MDA-MB-231 breast cancer cells. It arrested the cell cycle in the G1 phase and decreased CDK4 activity, increasing p53 tumor suppressor protein activity. It reduced the ATP levels of MCF-7 and MDA-MB-231 cells, induced apoptosis through both signaling pathways and inhibited invasion in both tumor lines. Furthermore, the combination of the extract with the antineoplastic drugs cisplatin and paclitaxel enhanced the cytotoxic effect, making EAB a potential adjuvant in the treatment of breast cancer.

## Figures and Tables

**Table 1:** Identified compounds in EAB by UPLC-Q-TOF/MS^E^.

Identification	Tr	Formula	Measured *m*/*z*	Mass Error (ppm)	Fragmentation
Gallic acid	2.38	C_7_H_6_O_5_	169.0000	−6.6	125.02269, 109.0276, 125.0230, 137.023
Epigallocatechin	3.65	C_15_H_14_O_7_	305.0667	1.6	139.0389, 165.0183, 167.033
3,4-Dihydroxybenzoic acid	3.77	C_7_H_6_O_4_	153.0230	−4.0	109.0276, 108.0198
Gallic acid 4-O-(6-galloylglucoside)	4.00	C_20_H_20_O_14_	483.0800	5.8	125.023, 169.0136, 211.0248, 313.0562
Catechin	4.06	C_15_H_14_O_6_	289.0716	1.3	123.0437, 125.0231, 203.0710, 245.0823
Epicatechin	4.17	C_15_H_14_O_6_	289.0721	1.4	109.0276, 123.0437, 161.0235
Procyanidin B2	4.31	C_30_H_26_O_12_	577.1357	1.0	161.0231, 245.0834, 289.0713, 407.0771
Epigallocatechin gallate	4.92	C_22_H_18_O_11_	457.7900	3.5	125.023, 169.0136, 287.0567, 305.0671
Gallocatechin 3-gallate	5.02	C_22_H_18_O_11_	457.0798	4.9	125.0231, 169.0136, 271.60, 289.0722
Quercetin-3-O-arabinoglucoside	5.12	C_26_H_28_O_16_	595.1308	1.4	300.01261, 271.0249, 255.0291
Quercetin-3-O-glucoside	5.38	C_21_H_19_O_12_	463.0876	0.4	300.0273, 285.0391
Ellagic acid	5.41	C_14_H_6_O_8_	300.9999	3.4	145.0283, 283.995
Epicatechin-3-O-gallate	5.45	C_22_H_18_O_10_	441.0912	2.7	125.023, 169.0136, 289.0722
Quercitrin	5.74	C_21_H_20_O_11_	447.0935	2.4	271.0274, 300.0815, 301.0350
Phloridzin	5.96	C_21_H_24_O_10_	435.1302	1.3	125.0228, 167.0340, 273.0767

**Table 2:** Cell cycle arrest in the G1 phase with EAB treatment in the MCF-7 and MDA-MB-231 cell lines.

Treatment/ Phases	MCF-7			MDA-MB-231		
	G1	S	G2	G1	S	G2
Control	51.7 ± 3.04	3.7 ± 0.21	44.6 ± 1.30	56.7 ± 0.28	1.51 ± 0.12	41.75 ± 0.07
Starvation	64 ± 4.38	0.15 ± 0.11	35.85 ± 3.17	58 ± 0.49	5.65 ± 0.15	36.35 ± 3.87
Cisplatin 13/13.65 μg/mL	19.85 ± 3.46	79.4 ± 3.53	0.75 ± 0.07	16.2 ± 0.14	82.8 ± 0.14	1 ± 0
EAB 500 μg/mL	84.05 ± 4.03	8.33 ± 0.73	7.35 ± 0.73	76.2 ± 2.26	8.3 ± 0.08	15.3 ± 0.06
EAB 1000 μg/mL	82.15 ± 7.61	0 ± 0	17.85 ± 0.56	82.85 ± 0.63	15.8 ± 1.13	1.36 ± 0.01

**Table 3:** Molecular docking between gallic acid, 3,4-dihydroxybenzoic acid, catechin and epicatechin compounds and proteins involved in necroptosis, cell invasion and the cell cycle.

Energy (Kcal/mol)								
Protein	Gallic Acid	Epigallocatechin	3,4-Dihydroxybenzoic Acid	Catechin	Epicatechin	Epigallocatechin Gallate	Gallocatechin 3-Gallate	Epicatechin-3-O-Gallate
ALDH1A3	−14.03	−6.96	−13.77	−6.76	−8.01	−0.27	−1.77	−1.85
IGF-R1	−13.79	-	−14.2	−2.37	−3.44	-	-	-
CDK1	−14.84	−3.00	−12.02	−3.52	−5.35	-	-	−0.35
CDK2	−10.43	-	−11.59	−3.21	−0.96	-	-	-
CDK4	−11.98	−0.31	−11.21	−7.51	−1.51	-	-	-
P53	−15.29	−0.93	−14.98	−6.79	−3.16	-	-	−1.2
TNFR1	−9.44	-	−9.45	-	-	-	-	-
MLKL	−9.51	-	−10.82	-	-	-	-	-
MMP2	−17.47	−3.41	−18.72	−3.34	−3.73	-	-	−3.57
MMP9	−18.26	−3.53	−16.25	−4.67	−5.95	-	-	-
E-cadherin	−10.98	-	−9.73	−0.45	−1.08	-	-	-
N-cadherin	−15.56	−1.94	−15.92	−2.29	−5.38	−0.25	−2.07	-

**Table 4:** Effect of the extracts in combination with cisplatin and paclitaxel on the viability of the MCF-7 and MDA-MB-231 cell lines in a 2D model.

Extract or Cisplatin/Combination	MCF-7	MDA-MB-231	Extract or Paclitaxel/ Combination	MCF-7	MDA-MB-231
	Cell Viability (%)			Cell Viability (%)	
Control	100	100	Control	100	100
½ IC_50_ EAB	72	73	½ IC_50_ EAB	72	73
IC_50_ EAB	50	49	IC_50_ EAB	50	49
½ IC_50_ Cisplatin	72	74	½ IC_50_ Paclitaxel	73	75
IC_50_ Cisplatin	49	49	IC_50_ Paclitaxel	52	50
½ IC_50_ EAB + ½ IC_50_ Cisplatin	68	67	½ IC_50_ EAB +½ IC_50_ Paclitaxel	43	41
½ IC_50_ EAB + IC_50_ Cisplatin	35	29	½ IC_50_ EAB + IC_50_ Paclitaxel	33	35
IC_50_ EAB + ½ IC_50_ Cisplatin	43	41	IC_50_ EAB + ½ IC_50_ Paclitaxel	41	38
IC_50_ EAB + IC_50_ Cisplatin	25	31	IC_50_ EAB + IC_50_ Paclitaxel	14	25

$Cisplatin\;{IC}_{50}$ in MCF-7:13 ± 0.5 µg/mL; $Cisplatin\;{IC}_{50}$ in MDA-MB-231: 13.65 ± 0.1 µg/mL; $Paclitaxel\;{IC}_{50}$ in MCF-7: 6.1 ± 0.04 µg µg/mL; $Paclitaxel\;{IC}_{50}$ in MDA-MB-231: 0.96 ± 0.05 µg/mL; $EAB\;{IC}_{50}$ in MCF-7: 49.3 ± 0.09 µg/mL; $EAB\;{IC}_{50}$ in MDA-MB-231: 3.7 ± 0.04 µg/mL.

**Table 5:** Effect of the extracts in combination with cisplatin and paclitaxel on the viability of the MCF-7 and MDA-MB-231 cell lines in a 3D model.

Extract or Cisplatin/Combination	MCF-7	MDA-MB231	Extract or Paclitaxel/ Combination	MCF-7	MDA-MB231
	Cell Viability (%)			Cell Viability (%)	
Control	100	100	Control	100	100
½ IC_50_ EAB	73	72	½ IC_50_ EAB	72	74
IC_50_ EAB	50	49	IC_50_ EAB	51	48
½ IC_50_ Cisplatin	76	75	½ IC_50_ Paclitaxel	74	74
IC_50_ Cisplatin	52	48	IC_50_ Paclitaxel	50	50
½ IC_50_ EAB + ½ IC_50_ Cisplatin	66	63	½ IC_50_ EAB + ½ IC_50_ Paclitaxel	65	60
½ IC_50_ EAB + IC_50_ Cisplatin	35	45	½ IC_50_ EAB + IC_50_ Paclitaxel	34	38
IC_50_ EAB + ½ IC_50_ Cisplatin	31	42	IC_50_ EAB + ½ IC_50_ Paclitaxel	31	43
IC_50_ EAB + IC_50_ Cisplatin	25	28	IC_50_ EAB + IC_50_ Paclitaxel	24	30

$Cisplatin\;{IC}_{50}$ in MCF-7:18.9 ± 0.02 µg/mL; $Cisplatin\;{IC}_{50}$ in MDA-MB-231: 20 ± 0.1 µg/mL; $Paclitaxel\;{IC}_{50}$ in MCF-7: 17.34 ± 0.2 µg/mL; $Paclitaxel\;{IC}_{50}$ in MDA-MB-231: 5.93 ± 0.05 µg/mL; $EAB\;{IC}_{50}$ in MCF-7: 375 ± 0.3 µg/mL; $EAB\;{IC}_{50}$ in MDA-MB-231: 135 ± 0.1 µg/mL.

## Data Availability

Data are available upon request to the corresponding author.

## Supplementary Materials

The following supporting information can be downloaded at https://www.mdpi.com/article/10.3390/ijms26125672/s1.
