# Supplementary material for: Ballota hirsuta Benth Arrests the Cell Cycle, Induces Apoptosis and Inhibits the Invasion of MCF-7 and MDA-MB-231 Cell Lines in 2D and 3D Models
Source: Int J Mol Sci. 2025 Jun 13;26(12):5672. doi: 10.3390/ijms26125672 (PMC12193204; doi:10.3390/ijms26125672)
Supplement: Supplementary file 1 [file ijms-26-05672-s001.zip › ijms-3657147-supplementary.pdf]

Table S1. Quantification of compounds in crude extract by HPLC.

Compound	μg/g dry sample
Catechin	4086.73
Epicatechin	1153.83
Phloridzin	175.56
Ellagic acid	81
3,4-dihydroxybenzoic acid	22.52
Gallic acid	15.04

Table S2. Molecular docking between gallic acid, 3,4-dihydroxybenzoic acid, catechin and epicatechin compounds and proteins involved in necroptosis, cell invasion and cell cycle.

GALLIC ACID							
Protein	Energy (Kcal(mol)	Interaction					
		Hydrogen Bonding	Distance (Å)	Polar	Distance (Å)	Hydrophobic interactions	Distance (Å)
ALDH1A3	-14.04	THR B:140<br>LEU B: 471<br>ALA B: 473	3.84<br>4.46<br>3.79	Pi Alkyl<br>LEU B: 471	4.86	-	-
IGF-R1	-13.8	LYS A: 1003<br>GLY A: 1122<br>GLU A: 1050	5.5<br>4.01<br>4.41	-	-	Pi Alkyl:<br>LYS A: 1003<br>ALA A: 1001<br>MET A: 1112	5.8<br>5.5<br>5.57
CDK1	-14.84	ARG A: 180<br>ALA A: 273<br>ASP A: 271<br>GLU A: 230<br>Carbon hidrogen bond<br>PRO A: 272	3.86<br>3.71<br>3.8<br>3.34<br><br>3.92	-	-	Pi alkyl:<br>PRO A: 272	4.99
CDK2	-10.44	LEU A: 124	3.85	-	-	-	-
CDK4	-11.98	CYS B: 135<br>GLU B: 56	4.35<br>4.36	Pi Donor hydrogen Bond:<br>VAL B: 137	4.3	-	-
P53	-15.29	ARG C: 390<br>GLU A: 373	4.27<br>4.05	Pi Donor hydrogen Bond:<br>GLN C: 394	5.19	Pi Alkyl:<br>MET A: 369<br>ARG C: 390	5.12<br>6.37
TNFR1	-9.45	TYR C: 115<br>PRO A: 100<br>SER B. 99<br>ARG A:98<br>GLU C: 116	5.64<br>4.6<br>4.57<br>5.93<br>4.57	Pi cation:<br>GLU A: 116<br>Pi anion:<br>ARG A:98	7.27<br>6.79	-	-

				Pi Donor Hydrogen Bond<br>GLN A:101	5.18		
MLKL	-18.27	ARG A: 370<br>GLU A: 293<br>carbon hydrogen bond<br>ASP A: 369	5.89<br>3.96	-	-	Pi Alkyl<br>ARG A: 370	5.65
MMP2	-17.47	ALA A:422<br>ILE A: 424<br>PRO A: 417	3.74<br>5.73<br>4.76	-	-	Pi Alkyl:<br>Leu A: 420<br>ALA A: 422	5.07<br>4.9
MMP9	-9.52	LYS A: 214<br>GLY A: 215<br>CYS A: 230<br>ALA A: 229	4.17<br>2.95<br>4<br>3.64	-	-	Pi Alkyl:<br>ALA A: 229<br>LYS A: 214	6.05<br>4.19
E-cadherin	-10.99	ASN A: 168<br>TYR A: 148<br>ASP A: 138<br>ALA A: 144	4.69<br>5.58<br>4.02<br>4.36	-	-	Pi alkyl:<br>ILE A: 146	4.14
N-cadherin	-15.56	ILE A: 424<br>GLU A: 89	4.64<br>4.24	Pi anión:<br>GLU A: 89	5.38	Pi Alkyl:<br>ALA A: 78<br>ILE A: 92<br>ALA A: 80	6.73<br>4.57<br>6.72
**3,4-DIHYDROXYBENZOIC ACID**							
ALDH1A3	-13.74	TRP B:180<br>GLU B: 411<br>Carbon Hydrogen Bond:<br>GLY B: 282	4.73<br>4.64<br>4.31	Pi Donor Hydrogen Bond:<br>TRP B: 282	2.73	Pi-Pi stacked:<br>Pi-Pi stacked:	5.84
IGF-R1	-14.2	-	-	Pi sigma:<br>MET A: 1112	5.59	ALA A: 1001	5.57
CDK1	-12.02	ARG B: 306<br>ARG A: 123<br>GLU B: 258<br>Carbon Hydrogen Bond:<br>HIS B: 303	6.14<br>5.76<br>4.14<br>3.37<br>5.8	-	-	-	-
CDK2	-11.59	LEU A: 124<br>ARG A: 122	3.86<br>6.18	-	-	Pi Alkyl:<br>ARG A: 150<br>LEU A: 124	5.01<br>4.05
CDK4	-11.21	TYR B: 191<br>GLU B: 56<br>Carbon Hydrogen Bond:<br>ARG B. 139	5.24<br>4.2<br>5.04<br>4.91<br>4.09	Pi Donor:<br>GLY B: 160	3.84	Pi Alkyl:<br>ARG B: 163	4.96

		VAL B: 137					
P53	-14.98	ARG C. 390<br>GLU A: 373	4.27<br>4.05	Pi Donor hydrogen Bond:<br>GLN C: 394	5.19	Pi Alkyl:<br>MET A. 369<br>ARG C: 390	5.12<br>6.37
TNFR1	-9.45	ARG A: 98<br>GLU C. 116<br>GLN B: 102	5.67<br>4.96<br>3.89	Pi anion:<br>ARG A: 98<br>Pi cation:<br>GLU A: 116	6.49<br>8.12	-	-
MLKL	-10.82	CYS A. 286<br>ARG A: 210	3.2<br>5.1	Pi anion:<br>GLU A: 293<br>Pi sigma:<br>LEU A: 209	6.8<br>4.47	-	-
MMP2	-18.72	PRO A: 417<br>ALA A: 422<br>LEU A: 420	5.1<br>3.65<br>5.5	Pi Donor hydrogen Bond:<br>THR A: 426	4.74	Pi Alkyl:<br>LEU A: 420	5.17
MMP9	-16.25	LYS A: 214<br>GLY A: 215<br>CYS A: 230	4.19<br>2.96<br>4.06	-	-	-	-
E-cadherin	-9.73	ASP A: 138<br>ASN A: 168	3.98<br>3.12	Pi Donor hydrogen Bond:<br>ILE A: 146	5.39	Pi Alkyl:<br>ALA A: 145	4.31
N-cadherin	-15.92	ASN A: 90	5.17	Pi anion:<br>GLU A: 89<br>Pi sigma:<br>ILE A: 92	5.56<br>4.49	-	-
**CATECHIN**							
ALDH1A3	-6.76	CYS B: 314<br>Carbon hydrogen Bond:<br>ASN B: 469	5.11<br>3.95	Pi sigma:<br>LEU B: 471<br>Pi-Pi stacked:<br>PHE B: 182	5.41	Alkyl:<br>Ile B: 132<br>Pi Alkyl:<br>PHE B: 308<br>CYS B: 313	4.78, 4.73<br>7.29<br>7.51
IGF-R1	-2.37	LEU A: 975<br>GLN A: 977<br>GLY A: 1122	4.67<br>5.2<br>3.24	Pi cation:<br>LYS A: 1003<br>Pi sulfur:<br>MET A: 1112	5.58<br>6.43	Pi Alkyl:<br>VAL A: 983	5.36
CDK1	-3.53	SER A: 182<br>SER A: 178<br>GLU A: 230<br>Carbon hydrogen Bond:<br>ARG A: 180	3.23	Pi catión:<br>ARG A: 180<br>PRO A: 272	4.9<br>4.5	Pi Alkyl:<br>PRO A: 184<br>PRO A: 272<br>ARG A: 180<br>Alkyl:<br>ARG A: 180<br>PRO A: 272	6.09<br>4.35<br>5.01<br>4.9<br>4.5
CDK2	-3.22	ARG A: 122	6.13	Pi sigma:		Pi alkyl:	

		ALA A: 149<br>TYR A: 180<br>LYS A: 178<br>Carbon hydrogen bond:<br>VAL A: 154	4.38<br>3.34<br>7.03	LEU A: 124<br>Pi-Pi T-shaped<br>TYR A: 180	4.96<br>4.87	ARG A: 150<br>Alkyl:<br>LEU A: 124	6.83<br>6.82
CDK4	-7.51	ASP B: 129	3.78	Pi-Pi T-shaped:<br>PHE B: 66	7.15	Pi Alkyl:<br>ALA B: 133	6.3
P53	-6.79	GLU B: 373<br>Carbon Hydrogen Bond:<br>GLU B: 366	3.63<br>3.56	-	-	Pi Alkyl:<br>MET B: 369	5.43<br>4.52
TNFR1	-	-	-	-	-	-	-
MLKL	-	-	-	-	-	-	-
MMP2	-3.35	GLU A: 515<br>THR A: 511<br>ALA A: 510	4.8, 4.55<br>4.61<br>4.63	-	-	Pi Alkyl:<br>PRO A: 154<br>Alkyl:<br>PRO A: 154	5.71<br>4.9
MMP9	-4.67	TYR A: 427<br>THR A: 258<br>CYS A: 230<br>Carbon hydrogen bond:<br>ALA A: 229	5.54<br>4.26<br>5.26<br>4.35	-	-	Pi Alkyl:<br>ALA A: 228<br>ALA A: 229<br>Alkyl:<br>ALA A: 229	5.14<br>4.34, 4.88<br>4.66
E-cadherin	-	-	-	-	-	-	-
N-cadherin	-2.3	GLU A: 129<br>THR A: 169<br>ASP A: 138<br>Carbon hydrogen Bond:<br>ALA A: 229	4.77<br>5.32<br>4.08<br>4.35	-	-	Pi - Alkyl:<br>ALA A: 228<br>ALA A: 229	5.14<br>4.34<br>4.88
			**EPICATECHIN**				
ALDH1A3	-8.01	LEU B: 471<br>THR B: 140<br>Carbon hydrogen Bond:<br>ARG B: 139	3.98<br>3.64<br>3.64	Pi sigma:<br>ILE B: 132	4.7	Pi Alkyl:<br>LEU B: 471<br>LEU B: 471<br>Alkyl:<br>LEU B: 471	6.05<br>4.76<br>5.58<br>4.76
IGF-R1	-3.44	SER A: 1059<br>ASP A: 1056	4.59<br>3.79	Pi sigma:<br>MET A: 1112	6.59	Pi Alkyl:<br>VAL A: 983<br>ALA A: 1001<br>Alkyl:<br>VAL A: 983	5.06<br>6.94<br>4.9
CDK1	-5.35	GLU A: 173<br>SER A: 182	4.12<br>3.84	Pi cation:<br>ARG A: 180	4.74<br>5.84	Alkyl:<br>PRO A: 272	4.6<br>5.64

		SER A: 233	3.67	Pi sigma:<br>LEU A. 234		ARG A: 180<br>Pi Alkyl:<br>PRO A: 184	7.06
CDK2	-0.97	ALA A: 149<br>LEU A: 124<br>ARG A: 122	3.35<br>3.71<br>6.45	-	-	Pi Alkyl:<br>ARG A: 150	4.98
CDK4	-1.51	ASP B: 129<br>Carbon hydrogen bond:<br>VAL A: 27	3.34<br>4.4	Pi sulfur:<br>CYS A: 8<br>Pi-Pi T-shaped:<br>HIS B: 68	7.07<br>6.19	-	-
P53	-3.16	GLU B: 373<br>LYS B: 370	3.38<br>3.01<br>4.11	Pi sigma:<br>MET B: 369	4.93	-	-
TNFR1	-	-	-	-	-	-	-
MLKL	-	-	-	-	-	-	-
MMP2	-3.74	GLU A: 515<br>TYR A: 216	4.71<br>4.94	Pi-Pi T-shaped:<br>TYR A: 425	5.54	-	-
MMP9	-3.96	PRO A: 219<br>ARG A: 221<br>VAL A: 217<br>GLU A: 130<br>THR A: 331	3.31<br>4.55<br>4.32<br>4.41<br>4.43	Pi Donor Hydrogen:<br>TYR A: 277	7.49	Pi Alkyl:<br>PRO A: 133<br>ALA A: 333<br>PRO A: 219<br>Alkyl:<br>PRO A: 272<br>PRO A: 133	5.28<br>6.11<br>5.06<br>6.36<br>5.91
E-cadherin	-1.09	ASN A: 168<br>ARG A: 167	3.59<br>4.6	Pi Donor hydrogen Bond:<br>GLN A: 197<br>Pi sigma:<br>ALA A: 145	4.73<br>4.08	Alkyl:<br>ALA A: 145	7
N-cadherin	-5.38	ASN A: 90<br>Carbon Hydrogen Bond:<br>GLU A: 89	5.57<br>5.32<br>3.89	Pi anion:<br>GLU A: 89<br>Pi sigma:<br>ILE A: 92	5.38<br>4.42	Pi Alkyl:<br>ALA A. 78<br>ALA A: 80<br>Alkyl:<br>ILE A. 92	6.43<br>6.54<br>6.13
**EPIGALLOCATECHIN**							
ALDH1A3	-6.97	LEU B: 471<br>THR B: 140<br>TRP B: 189<br>Carbon hydrogen Bond:<br>ARG B: 139	5.86<br>4.01<br>4.91<br>4.1	Pi sigma:<br>ILE B: 132	4.9	Pi Alkyl:<br>LEU B: 471<br>Alkyl:<br>LEU B: 471	5.09<br>5.59
IGF-R1	-	-	-	-	-	-	-
CDK1	-3	GLU A: 230	3.86	Pi anion:		Pi Alkyl:	

		SER A: 233<br>ASP A: 271	3.38<br>4.1	GLU A: 230	5.76	LEU A: 234<br>PRO A: 229<br>ALA A: 273	6.66<br>4.57<br>6.3
CDK2	-	-	-	-	-	-	-
CDK4	-0.31	ASP B: 129<br>LEU A: 6	3.31<br>3.49	Pi Donor hydrogeno:<br>CYS A: 8<br>Pi-Pi T-shaped:<br>HIS B: 68	6.22<br>6.31	Pi Alkyl:<br>ALA B: 133<br>ARG A: 26	5.92<br>5.91
P53	-0.93	GLU B: 373	3.31	Pi sigma:<br>MET B: 369	4.92	-	-
TNFR1	-	-	-	-	-	-	-
MLKL	-	-	-	-	-	-	-
MMP2	-3.11	TRY A: 425<br>GLU A: 515	4.93<br>4.73	Pi-Pi T-shaped:<br>TYR A: 425	5.59	Pi Alkyl:<br>PRO A: 514	5.02
MMP9	-3.54	THR A: 331<br>GLU A: 130<br>VAL A: 217<br>PRO A: 219<br>ARG A: 221	4.37, 4.75<br>4.43<br>4.31<br>3.28<br>4.53	Pi Donor hydrogen Bond:<br>TYR A: 277	7.44	Pi Alkyl:<br>PRO A: 133<br>ALA A: 333<br>Alkyl:<br>PRO A: 272<br>PRO A: 133	5.32<br>6.07<br>6.31<br>5.95
E-cadherin	-	-	-	-	-	-	-
N-cadherin	-1.94	ILE A: 24<br>ASN A: 90<br>Carbon Hydrogen Bond:<br>GLU A: 89	4.65<br>5.03<br>4.02	Pi anion:<br>GLU A: 89	5.3	Pi Alkyl:<br>ALA A: 78<br>ILE A: 92<br>ALA A: 80<br>Alkyl:<br>ILE A: 92	6.57<br>4.46<br>6.22<br>6.61
**EPIGALLOCATECHIN GALLATE**							
ALDH1A3	-0.27	ARG B: 139<br>THR B: 140<br>TRP B: 189<br>Carbon Hydrogen Bond:<br>LEU B: 471<br>ARG B: 139	5.73<br>3.97<br>4.89<br>3.75<br>3.6	Pi sigma:<br>IE B: 132	5.2	Pi Alkyl:<br>ARG B: 139	6.17
IGF-R1	-	-	-	-	-	-	-
CDK1							
CDK2	-	-	-	-	-	-	-
CDK4							

P53						-	-
TNFR1	-	-	-	-	-	-	-
MLKL	-	-	-	-	-	-	-
MMP2							
MMP9							
E-cadherin	-	-	-	-	-	-	-
N-cadherin	-0.25	ASN A: 90<br>GLU A: 89<br>SER A: 26<br>ARG A: 28	4.96<br>4.69<br>4.56<br>3.63	Pi anion:<br>GLU A: 89	5.44	Pi alkyl:<br>ILE A: 92<br>ALA A: 78<br>ALA A: 80	4.51<br>6.11<br>6.57
**GALLOCATECHIN 3- GALLATE**							
ALDH1A3	-1.77	THR B: 140<br>THR B: 315<br>Carbon hydrogen bond:<br>ARG B: 139<br>ASN B: 69	4.09<br>4.06<br>3.49<br>3.5	Pi-Pi T shaped:<br>PHE B: 308<br>Pi-Pi stacked:<br>PHE B: 308<br>Pi sigma:<br>LEU B: 471	5.57<br>5.86<br>3.83	Pi alkyl:<br>LEU B: 471<br>Alkyl:<br>ILE B: 132	5.34<br>4.99
IGF-R1	-	-	-	-	-	-	-
CDK1							
CDK2	-	-	-	-	-	-	-
CDK4							
P53						-	-
TNFR1	-	-	-	-	-	-	-
MLKL	-	-	-	-	-	-	-
MMP2							
MMP9							
E-cadherin	-	-	-	-	-	-	-
N-cadherin	-2.07	ARG A: 38<br>GLU A: 89<br>ASN A: 90	3.74<br>4.49<br>4.98	Pi anion:<br>GLU A: 89<br>Pi- Pi stacked:	5.24<br>4.62	Pi Alkyl:<br>ALA A: 80<br>ILE A: 92	6.58<br>4.53

				TRP A: 2		ALA A: 78	6.58
**EPICATECHIN 3-O-GALLATE**							
ALDH1A3	-1.86	TYR B: 472<br>ARG B: 139<br>Carbon Hydrogen Bond:<br>ARG B: 139	5.01<br>6.64<br><br>3.84	Pi cation:<br>ARG B: 139	7.76	Un favorable Donor - Donor:<br>ALA B: 473	2.96
IGF-R1	-	-	-	-	-	-	-
CDK1							
CDK2	-	-	-	-	-	-	-
CDK4							
P53	-1.2	GLN B: 393<br>GLN B: 394<br>GLN C: 358	4.04<br>4.12<br>4.4	Pi Donor Hydrogen Bond:<br>GLN B: 394<br>Pi sigma:<br>MET D: 369	4.75<br>6.83	-	-
TNFR1	-	-	-	-	-	-	-
MLKL	-	-	-	-	-	-	-
MMP2	-3.57	GLU A: 243<br>GLU A: 217<br>GLN A: 219<br>ASN A. 245<br>GLN A: 393	3.92<br>5.51<br>4.32<br>5.15<br>5.54	-	-	Pi Alkyl:<br>PRO A: 236<br>MET A: 373	5.95<br>6.37
MMP9							
E-cadherin	-	-	-	-	-	-	-
N-cadherin							
